# Elevated Serum Presepsin Identifies Herpes Simplex Virus-1 Reactivation in COVID-19 Patients

**DOI:** 10.3390/v17030357

**Published:** 2025-02-28

**Authors:** Patricia Mester, Dennis Keller, Claudia Kunst, Stephan Schmid, Sabrina Krautbauer, Martina Müller, Christa Buechler, Vlad Pavel

**Affiliations:** 1 Department of Internal Medicine I, Gastroenterology, Hepatology, Endocrinology, Rheumatology, Immunology, and Infectious Diseases, University Hospital Regensburg, 93053 Regensburg, Germany; patricia.mester@klinik.uni-regensburg.de (P.M.); dennis.keller@stud.uni-regensburg.de (D.K.); claudia.kunst@klinik.uni-regensburg.de (C.K.); stephan.schmid@klinik.uni-regensburg.de (S.S.); martina.mueller-schilling@klinik.uni-regensburg.de (M.M.); vlad.pavel@klinik.uni-regensburg.de (V.P.); 2 Institute of Clinical Chemistry and Laboratory Medicine, University Hospital Regensburg, 93053 Regensburg, Germany; sabrina.krautbauer@klinik.uni-regensburg.de

**Keywords:** COVID-19, presepsin, SARS-CoV-2, herpes simplex virus, bacterial superinfection

## Abstract

Presepsin, a cleaved peptide of soluble CD14, may become a promising biomarker for assessing disease severity and mortality in coronavirus disease 2019 (COVID-19). Patients with severe COVID-19 frequently develop bacterial and fungal superinfections, as well as herpes simplex virus-1 (HSV-1) reactivation, which may exacerbate disease progression. This study aimed to evaluate the impact of concomitant infections on serum presepsin levels. Serum presepsin levels were measured using an enzyme-linked immunosorbent assay (ELISA) in 63 patients with moderate COVID-19, 60 patients with severe disease, and 49 healthy controls. Correlations with procalcitonin and the presence of superinfections or HSV-1 reactivation were assessed. Consistent with previous studies, serum presepsin levels were the highest in patients with severe COVID-19 (*p* = 0.002 compared to patients with moderate disease). Within this group, non-survivors exhibited significantly elevated presepsin levels (*p* = 0.027). A positive correlation between presepsin and procalcitonin was observed in both moderate and severe COVID-19 cases. Patients with bacterial or fungal superinfections showed presepsin levels comparable to those without secondary infections. However, presepsin levels were markedly elevated in patients with HSV-1 reactivation (*p* = 0.002). After excluding patients with HSV-1 reactivation, presepsin levels no longer differed between moderate and severe COVID-19 cases, though they remained higher than in healthy controls (*p* < 0.001 for both comparisons). In conclusion, these findings suggest that elevated serum presepsin levels in severe COVID-19 are primarily driven by HSV-1 reactivation rather than bacterial or fungal superinfections.

## 1. Introduction

Severe acute respiratory syndrome coronavirus 2 (SARS-CoV-2) infection can lead to sepsis, yet the mechanisms determining why some patients develop mild to moderate symptoms while others progress to severe disease remain incompletely understood [1,2,3]. Identifying reliable prognostic biomarkers is crucial for early intervention and treatment optimization [4,5,6]. Increasing evidence supports presepsin as a potential diagnostic and prognostic marker of COVID-19 [7,8,9]. Cluster of differentiation (CD) 14 is a glycosylphosphatidylinositol-anchored membrane protein expressed mainly by monocytes and macrophages. CD14 binding to lipopolysaccharide (LPS), which forms a complex with LPS-binding protein, and subsequent association with toll-like receptor 4 initiate the immune response. Several different products of Gram-negative and Gram-positive bacteria that act as CD14 ligands have now been described [10]. Activation of monocytes and macrophages through CD14 is associated with proteolysis of CD14, and various soluble CD14 fragments are released into the circulation. One of them is presepsin, which is the N-terminal 64 amino acid fragment of CD14, and is generated under conditions of inflammation and oxidative stress [10]. Additionally, phagocytosis of bacteria by innate immune cells leads to CD14 internalization and degradation. CD14 internalization and enzymatic processing within the phagolysosome produces different CD14 fragments, and also increases presepsin levels [10,11]. The first 152 amino acids at the N-terminus of CD14 are sufficient to bind LPS and confer near-wild-type bioactivity in vitro. Presepsin is unlikely to interact with LPS, and its biological function remains largely undefined [12].

Beyond bacterial infections, presepsin elevation has been observed in non-infectious inflammatory conditions, including decompensated liver cirrhosis [13] and systemic lupus erythematosus [14]. Recent findings suggest that phagocytosis of neutrophil extracellular traps (NETs)—which express high levels of CD14—by monocytes and macrophages also contributes to presepsin production, challenging its specificity as a bacterial infection marker [15,16]. These insights underscore the broader role of presepsin in both infectious and non-infectious inflammatory processes, reinforcing the need for contextual interpretation when using presepsin as a biomarker in COVID-19 and other diseases.

In COVID-19 patients, plasma presepsin levels have been associated with disease severity, the need for mechanical ventilation, and mortality [8]. Serum presepsin levels were significantly higher in severe cases compared to those with mild symptoms [9]. However, among severe COVID-19 patients, survivors and non-survivors had similar presepsin levels [9], suggesting that presepsin’s association with mortality may be influenced by the inclusion of mild and moderate cases, where lower presepsin levels correspond to lower mortality risk [8]. A 2023 meta-analysis of 1373 COVID-19 patients concluded that plasma presepsin is a reliable biomarker for distinguishing mild from severe COVID-19 [7]. These findings reinforce the diagnostic value of presepsin in assessing disease severity, but suggest limited prognostic utility for predicting survival in severe cases.

In suspected sepsis patients, presepsin levels did not predict mortality. However, in this cohort, patients with confirmed bacterial infections—most commonly in the urinary tract, respiratory tract, or abdominal cavity—had elevated presepsin levels [17]. Sepsis patients with localized or systemic bacterial infections showed significantly higher presepsin levels compared to those without bacterial infections [18]. Furthermore, presepsin levels were higher in bacterial infections than in non-bacterial infections, reinforcing its potential as a biomarker for bacterial sepsis [18].

Between 23% and 28% of COVID-19 patients develop a bacterial superinfection within 48 h of intensive care unit admission [19,20]. A meta-analysis found that superinfections occurred in 24% of both hospitalized and non-hospitalized COVID-19 patients, with fungal and viral superinfections accounting for 8% and 4% of cases, respectively [21]. COVID-19 is also associated with herpes simplex virus 1 (HSV-1) reactivation [22]. An Italian study reported HSV reactivation in ~30% of invasively ventilated COVID-19 patients [23]. Superinfections worsen disease progression and increase mortality risk [21,24,25].

Whether bacterial superinfections contribute to further increases in serum presepsin levels in severe COVID-19 remains insufficiently studied. Additionally, to our knowledge, serum presepsin levels have not been measured in patients with HSV-1 reactivation, highlighting an important gap in research. Bacterial superinfection and HSV-1 reactivation are established risk factors for severe COVID-19 outcomes [21,24,25]. Therefore, we investigated whether serum presepsin levels are elevated in patients with HSV-1 reactivation and/or bacterial superinfection, both of which are linked to more severe COVID-19 disease [22,24,26].

## 2. Materials and Methods

### 2.1. Study Cohort

Blood samples were obtained from adult patients diagnosed with SARS-CoV-2 infection between April 2020 and January 2024. More than 90% of the blood samples were collected between April 2020 and June 2021. Serum was prepared and stored in aliquots at −80 °C. The majority of our patients had not yet received their SARS-CoV-2 vaccinations, which were first administered in Germany on 26 December 2020. COVID-19 patients were treated according to the guidelines of the German Federal Joint Committee and the European Medicines Agency. Remdesivir and dexamethasone were part of COVID-19 treatment in Germany, and all patients received heparin to prevent blood clots [27].

Sixty-three patients reported fever, tachycardia, dyspnea, and fatigue. These patients were included in the “moderate” COVID-19 group, as they met the criteria for systemic inflammatory response syndrome (SIRS) [28,29,30] and did not require admission to the intensive care unit. In the intensive care unit, 60 patients received treatment after developing septic shock. According to the NIH COVID-19 severity classification, these patients were classified as “severe” [29,30,31,32].

### 2.2. Measurement of Serum Presepsin Levels

The ELISA from MyBioSource.com (San Diego, CA, USA; catalog number: MBS456747; intra-assay precision CV < 10%, inter-assay precision CV < 12%) was used to measure the levels of presepsin in serum diluted 1:10. Each sample was assayed twice, and the average results were used. According to the package insert of the PATHFAST Presepsin kit, plasma samples are stable for 9 months at −20 °C or lower. There are no studies investigating the stability of presepsin in serum stored for a longer time [33,34].

### 2.3. Microbiological Tests and Bronchoalveolar Lavage

Microbiological diagnoses of blood stream infections were performed at the Institute of Clinical Microbiology and Hygiene at the University Hospital of Regensburg according to accredited laboratory standards. In brief, aerobic and lytic anaerobic blood culture bottles were routinely incubated for five days using the automated BD BACTEC FX blood culture system (Becton Dickinson, Heidelberg, Germany). Automatic detection of positive blood culture bottles was followed by Gram staining and subculture on standard solid growth media (chocolate blood, fresh blood, MacConkey agar) under a laminar flow hood. Subcultures were incubated overnight under standard aerobic or anaerobic conditions. Bacteria and yeasts were identified by matrix-assisted laser desorption ionization–time of flight mass spectrometry (Bruker Daltonics, Bremen, Germany) [35].

PCR was used to identify HSV-1 in the bronchoalveolar lavage samples. Bronchoalveolar lavage was performed during bronchoscopy in the intensive care unit. To perform bronchoalveolar lavage, the bronchoscope was inserted into the selected bronchopulmonary segment, selected from the computed tomography appearance, and 20 mL of saline was instilled and then withdrawn by negative suction pressure, and collected in specimen containers. The samples were immediately taken to the laboratory for analysis.

DNA was isolated from bronchoalveolar lavage using the EZ1 Virus Mini Kit v2.0 and the EZ1 Advanced XL system (Qiagen, Hilden, Germany) according to the manufacturer’s instructions. HSV-1 DNA was amplified using primers 5′-TCCTSGTTCCTMACKGCCTCCC-3′ and 5′-GCAGICAYACGTAACGCACGCT-3′ and TaqMan-HSV-1gG1-Probe (VIC-CGTCTGGACCAACCGCCACACAGGT-TAMRA) was used for detection (Metabion, Planegg, Germany). HSV-1 qPCR was performed on a StepOnePlus System (Applied Biosystems, Waltham, MA, USA). Plasmids containing HSV-1 sequences were used as standards for quantification. All precautions of accredited laboratories, particularly separate rooms for DNA extraction, amplification, and detection, were taken to avoid any cross-contaminations. All samples were tested in two replicates. Positive single values < 100 copies/mL of one replicate were considered negative. DNA copies were calculated as mean of two positive replicates. As lower limit of detection for the assay, 300 copies/mL were determined [36]. The number of HSV-1 copies was 3.6 × 10^7^ (600 − 1 × 10^11^)/L and was determined at the Institute of Clinical Microbiology and Hygiene at the University Hospital of Regensburg.

### 2.4. Routine Laboratory Testing

Serum levels of C-reactive protein (CRP) were measured using an advanced immunoturbidimetric assay technique, while interleukin-6 and procalcitonin concentrations were assessed using electrochemiluminescence immunoassays. Lactate dehydrogenase facilitates the conversion of L-lactate to pyruvate, resulting in the production of NADH, which is quantified via photometric analysis. The enzymatic oxidation of L-lactate by lactate oxidase yields pyruvate and hydrogen peroxide, the latter of which is detected using colorimetric methods. The activity of alkaline phosphatase is evaluated by the hydrolysis of p-nitrophenyl phosphate into phosphate and p-nitrophenol, both of which can be measured photometrically. The concentrations of ferritin are determined using an electrochemiluminescence immunoassay that employs ferritin-specific antibodies linked to ruthenium or biotin. The Cobas Pro analyzer, in conjunction with the relevant assays from Roche (Penzberg, Germany), was employed to assess the aforementioned parameters. These analyses were conducted at the Institute of Clinical Chemistry and Laboratory Medicine at the University Hospital Regensburg.

### 2.5. Statistical Analysis

Data are presented as box plots displaying the median, first and third quartiles, and minimum and maximum values. Outliers are marked with asterisks (presepsin levels > 3.0× the interquartile range) and circles (presepsin levels > 1.5× the interquartile range). The median, minimum, and maximum values are provided in the tables. Statistical analyses were performed using IBM SPSS Statistics 26.0 (IBM Corp. Released 2019. IBM SPSS Statistics for Windows, Version 26.0. Armonk, NY, USA: IBM Corp.). Non-parametric tests were applied, as the Shapiro–Wilk and Kolmogorov–Smirnov tests confirmed that serum presepsin levels were not normally distributed (*p* < 0.001 for both). Significance was assessed using the chi-square test, receiver operating characteristic (ROC) curve analysis, Mann–Whitney U test, Kruskal–Wallis test, and Spearman’s correlation. A *p*-value < 0.05 was considered significant.

## 3. Results

### 3.1. Serum Presepsin Levels of Healthy Controls and Patients with COVID-19

This study included 63 patients with moderate COVID-19 and 60 patients with severe disease. Serum from 49 healthy controls was also analyzed. The control cohort consisted of 24 males and 25 females, with a median age of 56 years (range: 24–81). Age and sex distribution were comparable between patients and controls.

Compared to those with moderate COVID-19, patients with severe disease exhibited elevated levels of procalcitonin, lactate dehydrogenase, ferritin, and C-reactive protein (CRP) (Table 1). In contrast, alkaline phosphatase and interleukin-6 levels did not differ significantly between the two patient groups (Table 1). Additionally, patients with severe COVID-19 had a higher body mass index (BMI) and increased counts of neutrophils, basophils, monocytes, and immature granulocytes. However, eosinophil and lymphocyte counts remained unchanged between the two groups (Table 1).

Serum presepsin levels did not differ by gender in either the moderate (*p* = 0.793) or severe (*p* = 0.468) COVID-19 groups. There was no correlation between presepsin levels and BMI in patients with moderate disease (r = −0.107, *p* = 0.560), while a significant negative correlation was observed in severe cases (r = −0.338, *p* = 0.011). Additionally, presepsin levels correlated positively with age in both moderate (r = 0.346, *p* = 0.006) and severe (r = 0.374, *p* = 0.004) disease.

In patients with moderate COVID-19, blood samples were collected 1 to 16 days after hospital admission (median: 3 days), while in patients with severe COVID-19, blood samples were collected 1 to 10 days after admission (median: 4 days). The timing of blood collection showed no correlation with serum presepsin levels in either the moderate (r = 0.113, *p* = 0.487) or severe (r = 0.099, *p* = 0.451) groups. Additionally, serum CRP, procalcitonin, lactate dehydrogenase, ferritin, and immune cell counts did not correlate with the day of blood collection in either cohort (*p* > 0.05 for all).

**Figure 1:**
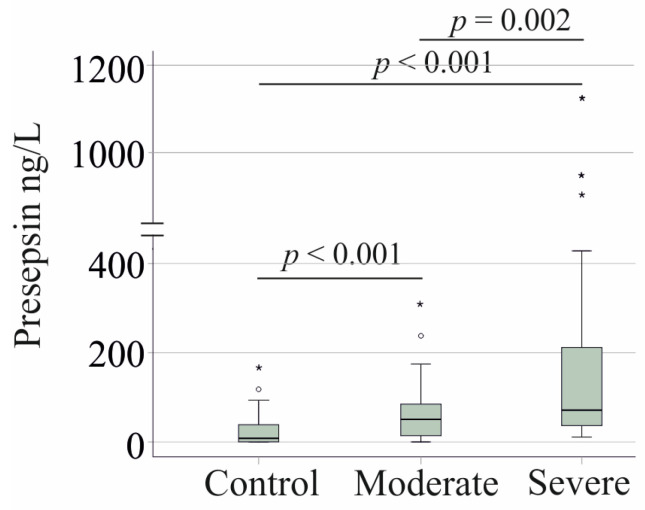
Serum presepsin levels of healthy controls and patients with COVID-19. Serum presepsin levels of controls and patients with moderate and severe COVID-19. Outliers are indicated by circles (presepsin levels > 1.5× the interquartile range) and asterisks (presepsin levels > 3.0× the interquartile range). Statistical test: Kruskal–Wallis test.

Median serum presepsin levels were 8 ng/L (range: 0–168) in healthy controls, 51 ng/L (range: 0–310) in moderate cases, and 71 ng/L (range: 11–1127) in severe cases. Presepsin levels were lowest in the controls, increased in moderate COVID-19, and were highest in severe disease (Figure 1). These findings indicate that serum presepsin levels rise with increasing disease severity.

The area under the receiver operating characteristic curve (AUROC) for predicting severe versus moderate COVID-19 by presepsin was 0.656 (*p* = 0.003), demonstrating that serum presepsin is a valid biomarker for assessing disease severity.

### 3.2. Effect of Vasopressor Therapy and Dialysis on Serum Presepsin

Our cohort included six patients with moderate COVID-19 who required dialysis. Their serum presepsin levels were comparable to those of patients not requiring dialysis (Table 2). However, in severe COVID-19 cases, patients requiring dialysis or vasopressor therapy exhibited significantly higher serum presepsin levels than those who did not require these interventions (Table 2).

### 3.3. Correlation of Serum Presepsin Levels with Inflammation

Serum presepsin levels were positively correlated with procalcitonin in both moderate and severe COVID-19 cases. In severe cases, presepsin levels were also associated with CRP, interleukin-6, and eosinophil counts. A significant correlation between presepsin and immature granulocytes was observed exclusively in the moderate SARS-CoV-2 cases (Table 3).

### 3.4. Serum Presepsin Levels of Patients with Superinfections and HSV-1 Reactivation

Six patients with moderate COVID-19 in our cohort developed a bacterial superinfection. Their serum presepsin levels were comparable to those of patients without bacterial superinfection (*p* = 0.796). HSV-1 reactivation was not observed in moderate cases, and two patients had fungal infections.

**Figure 2:**
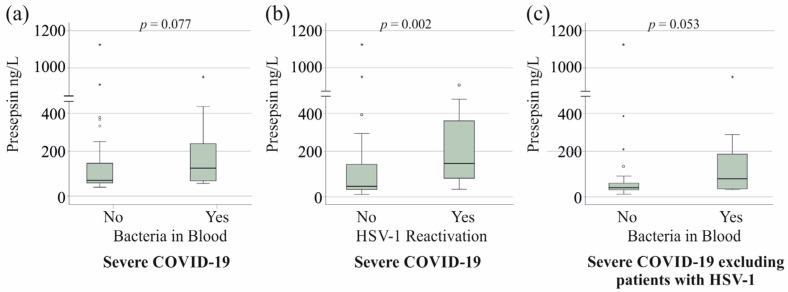
Serum presepsin levels of patients with severe COVID-19 and bacterial superinfection or herpes simplex virus 1 (HSV-1) reactivation. (**a**) Serum presepsin levels of patients with severe COVID-19 without (No) and with (Yes) bacteremia; (**b**) Serum presepsin levels of patients with severe COVID-19 without (No) and with (Yes) HSV-1 reactivation; (**c**) Serum presepsin levels of patients with severe COVID-19 without (No) and with (Yes) bacteremia after exclusion of the 20 patients with HSV-1. Outliers are indicated by circles (presepsin levels > 1.5× the interquartile range) and asterisks (presepsin levels > 3.0× the interquartile range). Statistical test: Mann–Whitney U test.

In severe COVID-19 cases, fungal infections did not influence serum presepsin levels (*p* > 0.05), nor did bacterial superinfection in 27 patients (Figure 2a). In contrast, HSV-1 reactivation in 20 severe cases was associated with significantly elevated presepsin levels (Figure 2b), with an area under the receiver operating characteristic curve (AUROC) of 0.747 (*p* = 0.002) for distinguishing between these groups. However, the HSV-1 DNA copy number did not correlate with serum presepsin levels (r = −0.126, *p* = 0.595).

Patients with HSV-1 reactivation clinically deteriorated and required escalation of ventilation parameters. Notably, there was no clinical manifestation of HSV-1 prior to COVID-19 in the medical history of the patients included in the study.

Notably, 11 patients with HSV-1 reactivation also had positive blood cultures for bacteria. Among the severe COVID-19 patients without HSV-1 reactivation, those with bacterial superinfection showed a trend toward higher serum presepsin levels (Figure 2c).

**Figure 3:**
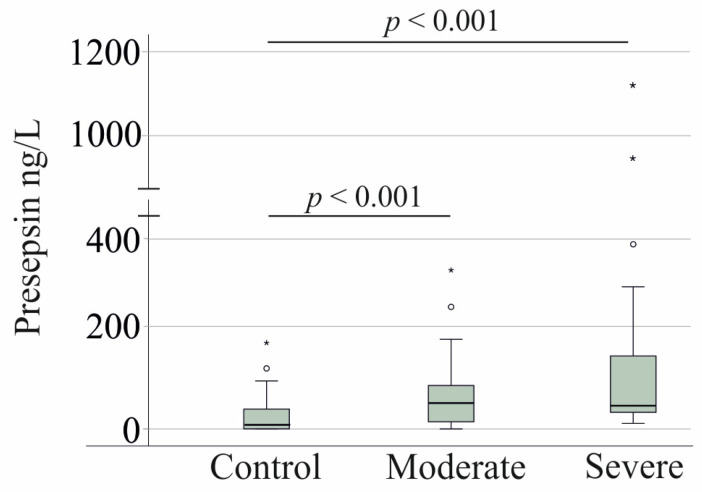
Serum presepsin levels of controls and patients with moderate and severe COVID-19 excluding the 20 patients with severe COVID-19 and HSV-1 reactivation. Outliers are indicated by circles (presepsin levels > 1.5× the interquartile range) and asterisks (presepsin levels > 3.0× the interquartile range). Statistical test: Kruskal–Wallis test.

After excluding patients with HSV-1 reactivation, serum presepsin levels remained lower in the healthy controls compared to the COVID-19 patients. In moderate cases, presepsin levels were 51 ng/L (range: 0–310), while in severe cases, they were 45 ng/L (range: 11–1127) (*p* = 0.278), showing no significant difference between the two groups (Figure 3).

After excluding the 20 patients with HSV-1 reactivation, serum presepsin levels were still correlated with CRP and procalcitonin (Table 4).

Of the seven patients with severe COVID-19 on dialysis, only one patient did not experience HSV-1 reactivation. Renal failure is associated with higher serum presepsin levels [37,38]. When patients on dialysis were excluded, those with severe COVID-19 and HSV-1 reactivation still had higher serum presepsin levels (*p* = 0.035). Among the 23 patients receiving catecholamines, serum presepsin levels were comparable to those in patients not receiving this treatment (*p* = 0.234) after excluding those with HSV-1 reactivation.

**Figure 4:**
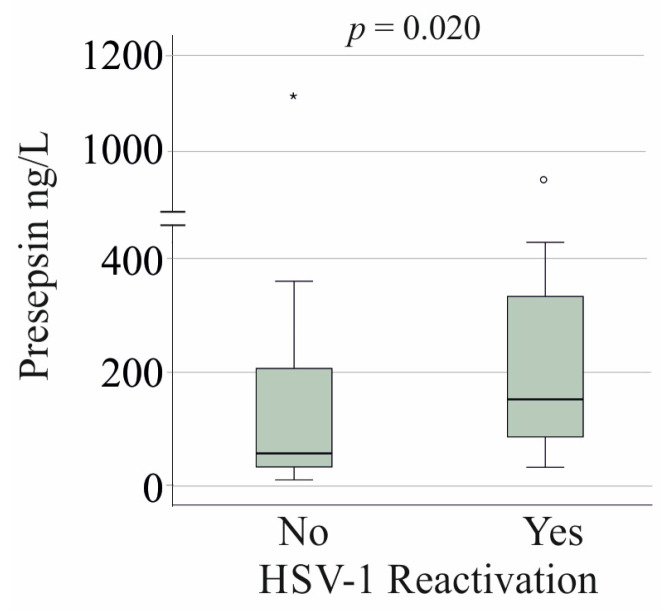
Serum presepsin levels of patients older than 51 years with severe COVID-19 and without (No) and with (Yes) herpes simplex virus-1 (HSV-1) reactivation. The age of the two groups was similar. Statistical test: Mann–Whitney U test. The small circle and the asterisk mark outliers.

Patients with HSV-1 reactivation were significantly older than those without (*p* < 0.001). However, when analyzing patients over 51 years of age (22 without HSV-1 reactivation and 19 with HSV-1 reactivation), the age distribution between the groups was similar (*p* = 0.190), yet serum presepsin levels remained elevated (Figure 4).

### 3.5. Serum Presepsin Levels and Survival

**Figure 5:**
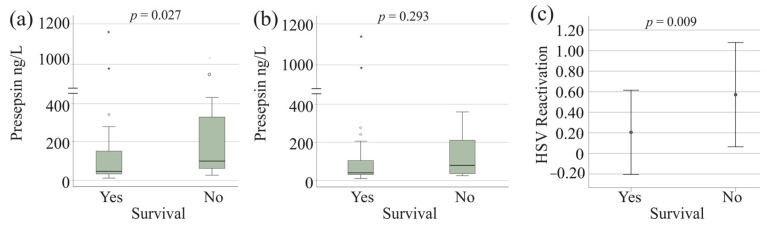
Serum presepsin levels and survival. (**a**) Serum presepsin levels of surviving and non-surviving patients with severe COVID-19; (**b**) Serum presepsin levels of surviving and non-surviving patients with severe COVID-19 after exclusion of patients with herpes simplex virus-1 (HSV-1) reactivation. Outliers are indicated by circles (presepsin levels > 1.5× the interquartile range) and asterisks (presepsin levels > 3.0× the interquartile range). Statistical test: Mann–Whitney U test; (**c**) Mean ± standard deviation of patients with HSV-1 reactivation (assigned number 1 and number 0 for those without HSV-1 reactivation for calculation) of surviving and non-surviving patients with severe COVID-19. Statistical test: chi-square test.

Among patients with severe COVID-19, 21 did not survive. Serum presepsin levels were higher in non-survivors (Figure 5a).

In patients without HSV-1 reactivation, 12 died, and presepsin levels did not differ significantly between survivors and non-survivors (*p* = 0.293). However, mortality was significantly higher in patients with HSV-1 reactivation (12 deaths in the 20 patients versus 9 deaths in the 40 patients without HSV-1 reactivation) compared to those without (*p* = 0.009) (Figure 5c).

## 4. Discussion

To our knowledge, this is the first study to analyze the relationship between HSV-1 reactivation, bacterial and fungal superinfections, and serum presepsin levels in COVID-19 patients. Our findings demonstrate that presepsin is specifically elevated in severe COVID-19 patients experiencing HSV-1 reactivation, whereas it does not differentiate between moderate and severe disease in the absence of HSV-1 reactivation. These results highlight presepsin as a distinct biomarker for HSV-1 reactivation in COVID-19, providing potential clinical value for patient stratification and targeted management.

A recent study reported serum presepsin levels of approximately 1100 g/L in patients with moderate COVID-19 and 1250 ng/L in those with severe disease [9]. In contrast, our cohort exhibited significantly lower presepsin levels, with median values of 51 ng/L (range: 0–310) in moderate cases and 71 ng/L (range: 11–1127) in severe cases. Levels between 20 and 600 ng/L were detected in patients with low-severity COVID-19 [7]. These differences underscore the variability in absolute presepsin levels across studies, likely due to differences in patient populations, disease definitions, or assay methodologies.

An earlier meta-analysis showed a mean difference of almost 450 ng/L between patients with low-severity and high-severity COVID-19 [7]. Presepsin levels were 2–6 times higher in severe COVID-19 compared to moderate cases. However, increases of only 150% have also been observed [7]. Another study reported an almost seven-fold increase in high-severity compared to low-severity COVID-19 patients [39]. In healthy controls and patients with mild COVID-19, presepsin levels were around 500 ng/L, rising to 1200 ng/L in moderate cases and peaking at 3500 ng/L in severe cases [39]. Absolute presepsin values measured by different ELISA assays vary widely, ranging from 20 to 500 ng/L in mild cases and from 60 to 3500 ng/L in severe disease [7]. This variability highlights the urgent need for assay standardization to establish presepsin as a reliable clinical biomarker for COVID-19 severity and associated complications. In order to carry out multicenter studies with the aim of establishing presepsin as a biomarker for clinical diagnostic purposes, standardized procedures for sample collection, sample storage, and analysis of presepsin need to be established.

In our cohort, serum presepsin levels correlated positively with CRP and procalcitonin, even after excluding patients with HSV-1 reactivation. In contrast, previous studies have reported a positive association with CRP but not with procalcitonin in COVID-19 patients [8,40]. Given that presepsin production is driven by inflammatory responses [7,10,15], its correlation with CRP aligns with expectations. However, the lack of a consistent correlation between presepsin and procalcitonin in earlier studies [8,40] remains unexplained, particularly since procalcitonin levels are also elevated in critically ill patients [41,42]. These findings suggest potential differences in the regulatory mechanisms governing presepsin and procalcitonin in severe infections and COVID-19, warranting further investigation.

CRP and procalcitonin are routinely used to detect infection or inflammation [43,44,45]. As we have shown in our work, CRP and procalcitonin were also correlated with disease severity in COVID-19 patients. CRP and procalcitonin can be helpful in suspecting infectious or inflammatory diseases, but these two markers are not specific for detecting the source of infection or distinguishing between specific pathologies [43,44,45]. Furthermore, as the serological values of these markers are influenced by non-infectious pathological conditions, such as cirrhosis or rheumatological diseases, these markers are not always reliable in detecting infection [46,47,48]. This is why additional biomarkers are needed for the assessment of disease severity and the early diagnosis of superinfections.

The presence of CD14 in circulating monocytes [49,50] suggests that white blood cell counts could influence serum presepsin levels. However, previous studies have found no correlation between presepsin and leukocyte counts in patients with community-acquired pneumonia [51] or COVID-19 [52]. Consistent with these findings, our analysis revealed no significant correlation between presepsin and monocyte or neutrophil counts. However, presepsin levels showed a modest positive association with immature granulocytes in moderate COVID-19 cases and with eosinophils in severe cases. This study reinforces previous findings that presepsin levels are largely independent of total leukocyte counts, while highlighting potential links to specific immune cell populations, warranting further investigation.

Patients requiring dialysis or vasopressor therapy, indicative of more severe disease, had higher serum presepsin levels than those not requiring these interventions. However, the association between vasopressor therapy and elevated presepsin levels disappeared after excluding patients with HSV-1 reactivation, suggesting that HSV-1 rather than hemodynamic instability may be driving presepsin elevation in these cases.

Notably, all but one dialysis patient had HSV-1 reactivation, reinforcing a potential link between renal dysfunction and HSV-1 in severe COVID-19. Previous population-based studies have identified underlying renal disease and older age as risk factors for HSV-1 reactivation [53]. Our findings further suggest that HSV-1 reactivation is closely associated with the need for dialysis in severe COVID-19, highlighting the importance of monitoring both renal function and viral reactivation in critically ill patients.

Presepsin levels increase in acute and chronic renal failure [37,38]. Patients on dialysis had plasma presepsin levels similar to those of patients with sepsis and normal renal function [38]. Presepsin is metabolized by the kidney, and impaired renal function leads to increased systemic levels [37,38]. HSV-1 reactivation was still associated with higher serum presepsin levels when patients with severe COVID-19 on dialysis were excluded. Moreover, patients with moderate COVID-19 on dialysis did not have higher serum presepsin levels, showing that the association between renal impairment and serum presepsin levels in patients with COVID-19 needs further study. However, the number of patients was small, and these findings require confirmation in larger cohorts.

Serum presepsin levels have been identified as a prognostic marker for mortality in critically ill patients [40]. In our cohort, non-survivors had higher presepsin levels than survivors, both in the entire cohort and among patients with severe COVID-19. However, after excluding patients with HSV-1 reactivation, this difference disappeared, suggesting that HSV-1 reactivation, rather than presepsin itself, drives the association with mortality. There were only 20 patients with HSV-1 reactivation, and these findings require confirmation in larger cohorts.

HSV-1 reactivation was linked to higher mortality in our cohort, consistent with recent findings [54]. These results indicate that presepsin levels alone may not be a direct predictor of mortality in severe COVID-19, but instead reflect the impact of HSV-1 reactivation on patient outcomes. This underscores the importance of considering viral reactivation when interpreting presepsin as a prognostic biomarker.

Serum presepsin levels were similar in severe COVID-19 patients with and without bacterial superinfection, contrasting with previous studies that reported higher presepsin levels in sepsis patients with bacterial infections [17,18]. However, after excluding patients with HSV-1 reactivation, presepsin levels in severe COVID-19 patients with bacterial superinfection showed an almost significant increase, aligning with findings in sepsis [17,18]. These results suggest that bacteremia is associated with elevated presepsin levels, which is consistent with prior research [17,18]. However, the increase observed in bacterial superinfection was modest compared to the marked elevation linked to HSV-1 reactivation. This underscores HSV-1 reactivation as the dominant factor driving presepsin elevation in severe COVID-19, emphasizing its potential as a biomarker for viral reactivation rather than bacterial infection.

Serum presepsin levels correlated positively with age, consistent with previous findings [8]. Patients with HSV-1 reactivation were significantly older than those without, which aligns with prior studies that identified older age as a risk factor for HSV-1 reactivation in COVID-19 [55].

In an age-matched cohort, HSV-1 reactivation remained independently associated with higher presepsin levels, indicating that age alone does not fully explain the presepsin increase. These findings suggest that both older age and HSV-1 reactivation are key contributors to elevated presepsin levels in severe COVID-19. This reinforces presepsin as a biomarker not only for disease severity but also for viral reactivation, particularly in older patients.

HSV-1 reactivation is common in patients with acute respiratory distress syndrome (ARDS) and prolonged mechanical ventilation [56]. In our cohort, nearly all severe COVID-19 patients required ventilation, and approximately 30% exhibited HSV-1 reactivation, consistent with an Italian study reporting HSV-1 reactivation in 30% of invasively ventilated COVID-19 patients [23]. Given this high prevalence, regular HSV-1 screening in mechanically ventilated patients is essential for early detection and timely antiviral therapy, potentially improving clinical outcomes in severe COVID-19.

Several studies have postulated that blood presepsin is an excellent marker for monitoring disease severity and outcome in patients with sepsis [7,18,40,57]. Older age, renal failure, and HSV-1 reactivation are associated with higher serum presepsin levels, and these confounders need to be addressed in future clinical trials. If these analyses identify presepsin as an independent biomarker in severe disease, this short protein may also be evaluated as a target for therapy. In order to do this, the physiological and pathophysiological roles of presepsin need to be clarified.

This study has some limitations. Laboratory values for the control group were not measured, but were assumed to be within the normal range, as all controls were healthy and had normal body weight. Additionally, because all participants were from Bavaria, Germany, the findings may not be fully generalizable to populations in other regions. The serum samples were collected over almost 4 years, and the data may be less reliable because of the long storage time. However, no data are available on the stability of presepsin levels during prolonged storage [33,34]. Moreover, more than 90% of our blood samples were collected within 14 months, showing that the storage time of most samples was not very different. Our study cohort was relatively small, and it was not possible to take into account all the confounding factors, such as renal dysfunction and older age. Considerable variations in the sampling time of patients’ sera preclude the consideration of short-term changes in presepsin levels. Most studies have suggested the usefulness of presepsin as a biomarker for disease severity using a single measurement at the time of admission. Presepsin decreased within 72 h after admission in patients with sepsis/septic shock and more severe cases had a slower decrease [57]. In our cohort, presepsin in the serum did not correlate with the time of blood collection.

Despite these limitations, the study provides valuable insights into presepsin as a biomarker for HSV-1 reactivation in COVID-19, highlighting the need for further research in diverse populations.

## 5. Conclusions

These findings highlight a novel aspect of presepsin biology, demonstrating its clinical relevance as an indicator of viral reactivation rather than disease severity. Elevated serum presepsin levels in severe COVID-19 are also driven by older age and may increase during renal dysfunction, both of which need to be considered in studies evaluating blood presepsin levels as a biomarker of disease severity.

## Figures and Tables

**Table 1:** Characteristics of patients with moderate and severe COVID-19 and controls. The use of superscript numbers indicates that laboratory values were not documented for the entire group of patients, but rather for a subset of patients. The median value, the minimum value, and the maximum value of each parameter are given. Statistical test: Mann–Whitney U test and chi-square test.

Parameter	Moderate COVID-19	Severe COVID-19
Males/Females	34/29	42/18
Age (years)	60 (22–83)	57 (31–83)
BMI (kg/m^2^)	26.3 (18.4–42.6) ^32^	29.4 (19.2–66.7) ^56; *p* = 0.008^
C-reactive protein (mg/L)	26 (0–222)	74 (1–367) *^p^* ^< 0.001^
Procalcitonin (ng/L)	90 (0–24,900)	240 (60–25,000) ^*p* < 0.001^
LDH (U/L)	224 (127–929) ^39^	378 (162–1534) ^*p* < 0.001^
AP (U/L)	96 (38–372) ^29^	99 (37–743)
Ferritin (ng/mL)	573 (32–4826) ^45^	1088 (77–21,976) ^60; *p* < 0.001^
Interleukin-6 (ng/L)	19 (4–265) ^37^	36 (3–1175)
Neutrophils (n × 10^9^/L)	4.05 (0.13–23.10)	8.18 (0.90–24.91) ^*p* < 0.001^
Basophils (n × 10^9^/L)	0.03 (0–0.21)	0.05 (0.01–0.17) *^p^* ^< 0.001^
Eosinophils (n × 10^9^/L)	0.08 (0–1.19)	0.04 (0–1.07)
Monocytes (n × 10^9^/L)	0.57 (0.07–2.52)	0.71 (0.03–2.21) *^p^* ^= 0.037^
Lymphocytes (n × 10^9^/L)	1.11 (0.09–57.83)	1.20 (0–75.95)
Immature granulocytes (n × 10^9^/L)	0.03 (0–1.38)	0.25 (0.04–2.92) ^*p* < 0.001^

Alkaline phosphatase: AP; body mass index: BMI; lactate dehydrogenase: LDH. The *p*-values are given in the table.

**Table 2:** Serum presepsin levels (ng/L) of patients on dialysis and vasopressor therapy in comparison to those who did not receive these treatments. The median value, the minimum value, and the maximum value of each parameter are given. The *p*-values are given in the table. Statistical test: Mann–Whitney U test.

Intervention/Drug	No	Yes	*p*-Value
Moderate COVID-19			
Dialysis (6 patients)	50 (0–310)	80 (11–238)	0.274
Severe COVID-19			
Dialysis (7 patients)	61 (11–1127)	246 (33–907)	0.039
Catecholamine (41 patients)	44 (11–360)	99 (21–1127)	0.016

**Table 3:** Spearman correlation coefficients for the correlation of serum presepsin levels with inflammatory parameters and immune cell counts. The *p*-values are given in the table. Statistical test: Mann–Whitney U test.

Inflammation Marker	Moderate COVID-19	Severe COVID-19
C-reactive protein	0.241	0.357 *^p^* ^= 0.005^
Procalcitonin	0.519 *^p^* ^< 0.001^	0.434 *^p^* ^= 0.001^
Interleukin-6	0.164	0.294 *^p^* ^= 0.023^
Ferritin	0.107	0.246
Neutrophils	0.187	0.035
Basophils	0.161	0.186
Eosinophils	0.139	0.310 *^p^* ^= 0.016^
Monocytes	0.092	−0.002
Lymphocytes	0.006	0.074
Immature granulocytes	0.305 *^p^* ^= 0.015^	−0.041

**Table 4:** Spearman correlation coefficients for the correlation of serum presepsin levels with inflammatory parameters in patients with severe COVID-19 after excluding the 20 patients with severe COVID-19 with HSV-1 reactivation. The *p*-values are given in the table.

Inflammation Marker	Severe COVID-19
C-reactive protein	0.399 *^p^* ^= 0.011^
Procalcitonin	0.361 *^p^* ^= 0.022^

## Data Availability

The data supporting the findings of this study are available within the article.
